# Anti-VEGF neutralizing antibody delays osteomucosal healing by reducing collagen formation in mice

**DOI:** 10.1038/s41598-025-20840-x

**Published:** 2025-10-22

**Authors:** Eun-Bin Bae, Moon-Young Kim, Suk Ji, In-Woo Cho, Soo Yeon Kim, Maryam Esmaeili, Sherry S. Baik, Sol Kim, Sotirios Tetradis, No-Hee Park, Yousang Gwack, Reuben H. Kim

**Affiliations:** 1 The Shapiro Family Laboratory of Viral Oncology and Aging Research, Center for the Health Sciences, UCLA School of Dentistry, 10833 Le Conte Ave, Los Angeles, CA 90095 USA; 2 Department of Oral and Maxillofacial Surgery, Dankook University, Cheonan, 31116 Republic of Korea; 3 Department of Periodontology, Institute of Oral Health Science, Ajou University School of Medicine, Suwon, 16599 Republic of Korea; 4 Department of Periodontology, Dankook University, Cheonan, 31116 Republic of Korea; 5 Section of Oral and Maxillofacial Radiology, UCLA School of Dentistry, Los Angeles, CA 90095 USA; 6 UCLA Jonsson Comprehensive Cancer Center, Los Angeles, CA 90095 USA; 7 Department of Physiology, UCLA David Geffen School of Medicine, Los Angeles, CA 90095 USA

**Keywords:** Anti-VEGF antibody, Bevacizumab, MRONJ, Periodontitis, Extraction, Healing, Oral diseases, Chronic inflammation, Bone

## Abstract

Medication-related osteonecrosis of the jaw (MRONJ) is a detrimental side effect in patients undergoing treatment with antiresorptive agents. The anti-angiogenic agent, bevacizumab (anti-VEGF antibody (Ab)), has also been reported to be associated with MRONJ. However, the role of anti-VEGF Ab in MRONJ development, especially under conditions of pre-existing inflammation, remains elusive. This study examined anti-VEGF Ab effects on bone necrosis and osteomucosal healing, with or without pre-inflammation. Forty mice received biweekly i.p. injections of anti-VEGF Ab (10 mg/kg) or saline (Veh). For the tooth extraction (TE) model (*n* = 10), maxillary first molars were extracted. For the ligature-induced periodontitis and tooth extraction (LIP-TE) model (*n* = 10), maxillary second molars were ligated with 5−0 silk for 8 weeks before extraction. Mice were euthanized after 3 weeks of post-extraction healing. In both TE and LIP-TE models, anti-VEGF Ab-treated mice showed delayed osteomucosal healing with diminished bone formation, lower CD31 and collagen III expression, and increased osteoclast numbers than Veh-treated mice. There was no significant difference in necrotic bone areas. IL-23- or IL-17-producing cell numbers remained unchanged in both Veh- and anti-VEGF Ab-treated mice. Anti-VEGF Ab delayed osteomucosal healing by reducing collagen production in the presence or absence of pre-inflammatory conditions, without causing bone necrosis. Our data suggest anti-VEGF Ab delays osteomucosal wound healing but does not cause bone necrosis alone.

## Introduction

Medication-related osteonecrosis of the jaw (MRONJ) is a rare but serious intraoral lesion that preferentially occurs in patients who have received anti-resorptive drugs such as bisphosphonates (BPs) and denosumab (Dmab, anti-RANKL antibody (Ab)). BPs and Dmab are potent bone remodeling inhibitors that are associated with MRONJ development by suppressing osteoclast function and differentiation, respectively^1^. Indeed, studies have shown that BPs or Dmab alone can induce MRONJ following tooth extraction, the process requiring proper bone remodeling for wound healing^2,3^. Nonetheless, the precise underlying mechanisms of MRONJ are still elusive.

American Association of Oral and Maxillofacial Surgeons (AAOMS) reported that the MRONJ pathophysiology is associated with bone remodeling inhibition, inflammation or infection, angiogenesis inhibition, innate or acquired immune dysfunction, and genetic factors^4^. Indeed, increasing lines of evidence suggest that inflammation and infection play additional key roles in MRONJ pathogenesis. A recent retrospective study with 3734 teeth in cancer patients receiving high dose anti-resorptive drugs demonstrated that apical lesions, periapical osteosclerosis, and local infection are risk factors that increase the likelihood of developing MRONJ^5^. Similarly, many animal models recapitulating inflammatory diseases including periodontitis or periapical periodontitis demonstrated that these pre-existing inflammatory diseases can further exacerbate bone necrosis^6–10^, underscoring the importance of inflammation and infection as critical risk factors for MRONJ development.

Angiogenesis inhibition is another risk factor that has also been shown to be associated with MRONJ development^11–14^. Anti-vascular endothelial growth factor (VEGF) neutralizing Ab such as bevacizumab (Bmab) is an angiogenesis inhibitor that was first developed and approved to be used in conjunction with standard chemotherapy to treat metastatic colorectal cancer^15^. Over the past 15 years, its indication has been broadened to other cancer treatments such as metastatic breast cancer, ovarian and cervical cancer, non-small-cell lung cancer, and renal cell carcinomas^16^. Bmab specifically binds to VEGF-A, but not VEGF-B or VEGF-C, at Gly88 residues and neutralizes its binding to VEGF receptors^17^, leading to inhibition of blood vessel formation. Lack of blood supply is a paramount etiological factor in other diseases that cause bone necrosis such as osteoradionecrosis of the jaw (ORN) after radiation therapy. As such, Bmab has been suggested to play a role in MRONJ.

Several studies examined the effect of anti-VEGF neutralizing Ab in inducing bone necrosis and found that anti-VEGF Ab delayed wound healing without causing bone necrosis^11,18^. However, it remains to be determined whether anti-VEGF Ab therapy can potentially induce bone necrosis in the presence of pre-existing inflammatory conditions such as periodontitis. In this study, we used the anti-VEGF Ab in both tooth extraction (TE) and ligature-induced periodontitis (LIP-TE) mouse models to examine the direct effect of anti-VEGF Ab in osteomucosal healing and investigate its underlying mechanisms.

## Results

### Anti-VEGF Ab diminished bone formation in the TE mouse model

**Fig. 1:**
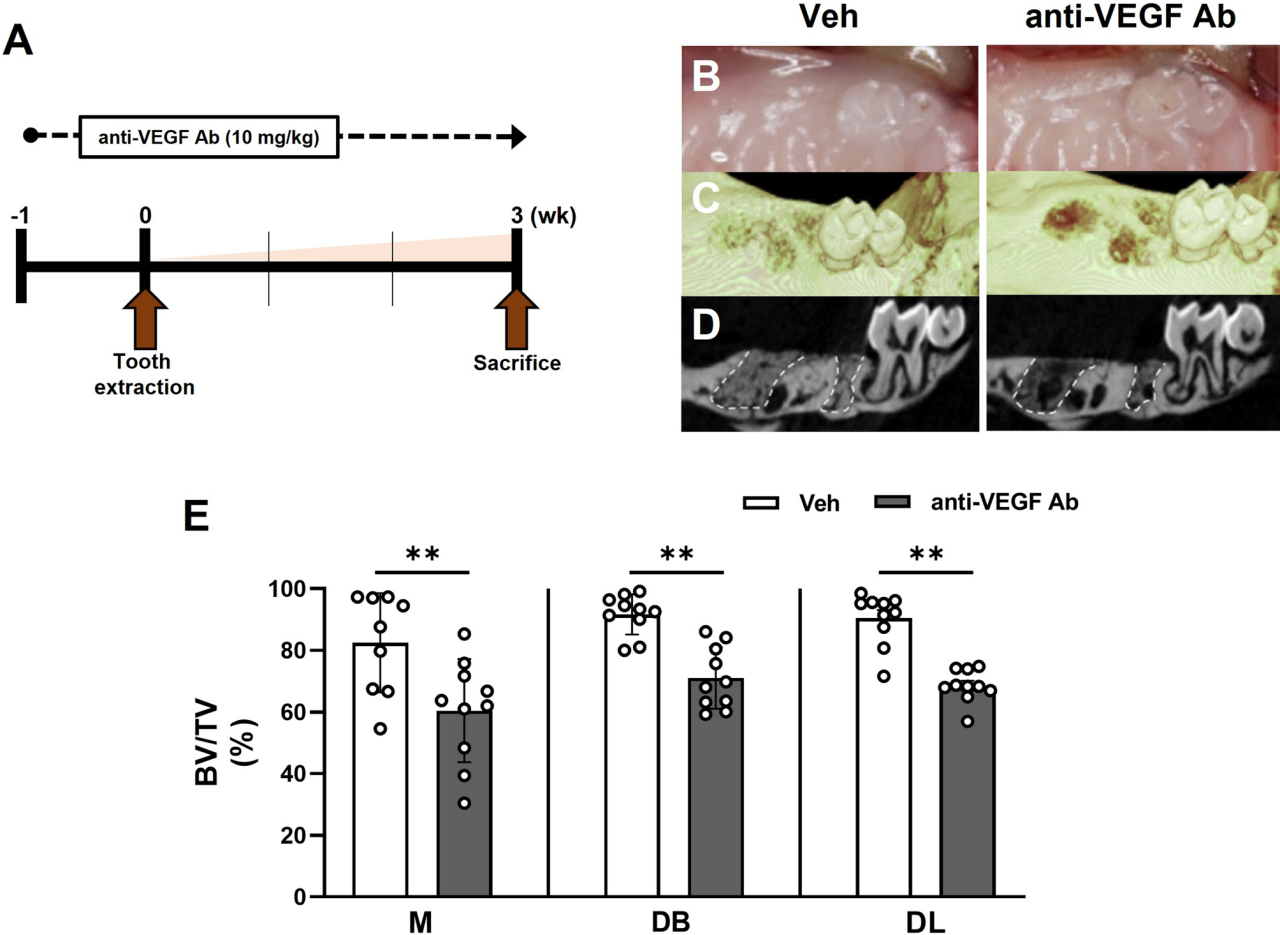
Anti-VEGF Ab diminished bone formation in the TE mouse model. (**A**) Timeline for TE model. (**B**) Occlusal views of the clinical photos and (**C**) 3D reconstructed µCT images in Veh- or anti-VEGF Ab-treated mice 3 weeks after extraction of first molars. (**D**) 2D sagittal views of the tooth extraction socket. (**E**) Quantification of bone volume fractions in the mesial (M), distobuccal (DB), or distolingual (DL) root areas following tooth extraction.

To examine the role of anti-VEGF Ab in osteomucosal healing after tooth extraction, we administered anti-VEGF Ab biweekly to the mice throughout the study period. One week after anti-VEGF Ab administration, first molars were extracted, and mice were allowed to heal for 3 weeks after which they were euthanized (Fig. 1A). In both vehicle (Veh)- and anti-VEGF Ab-treated groups, the soft tissues were completely healed (Fig. 1B), but tooth extraction sockets were less filled with new bone in the anti-VEGF Ab-treated mice (Fig. 1C). Sagittal sections of the extracted areas showed more radiolucency in the anti-VEGF Ab-treated groups when compared to the control Veh-treated group (Fig. 1D). Volumetric analysis of the extraction socket revealed reduced bone volume fraction (BV/TV) in all extracted sites in the anti-VEGF Ab-treated group when compared to the control Veh-treated group (Fig. 1E). These data indicate that anti-VEGF Ab delays bone formation in the tooth extraction sockets without compromising soft tissue closure.

### Anti-VEGF Ab suppressed vasculature and reduced type III collagen expression in the TE mouse model

**Fig. 2:**
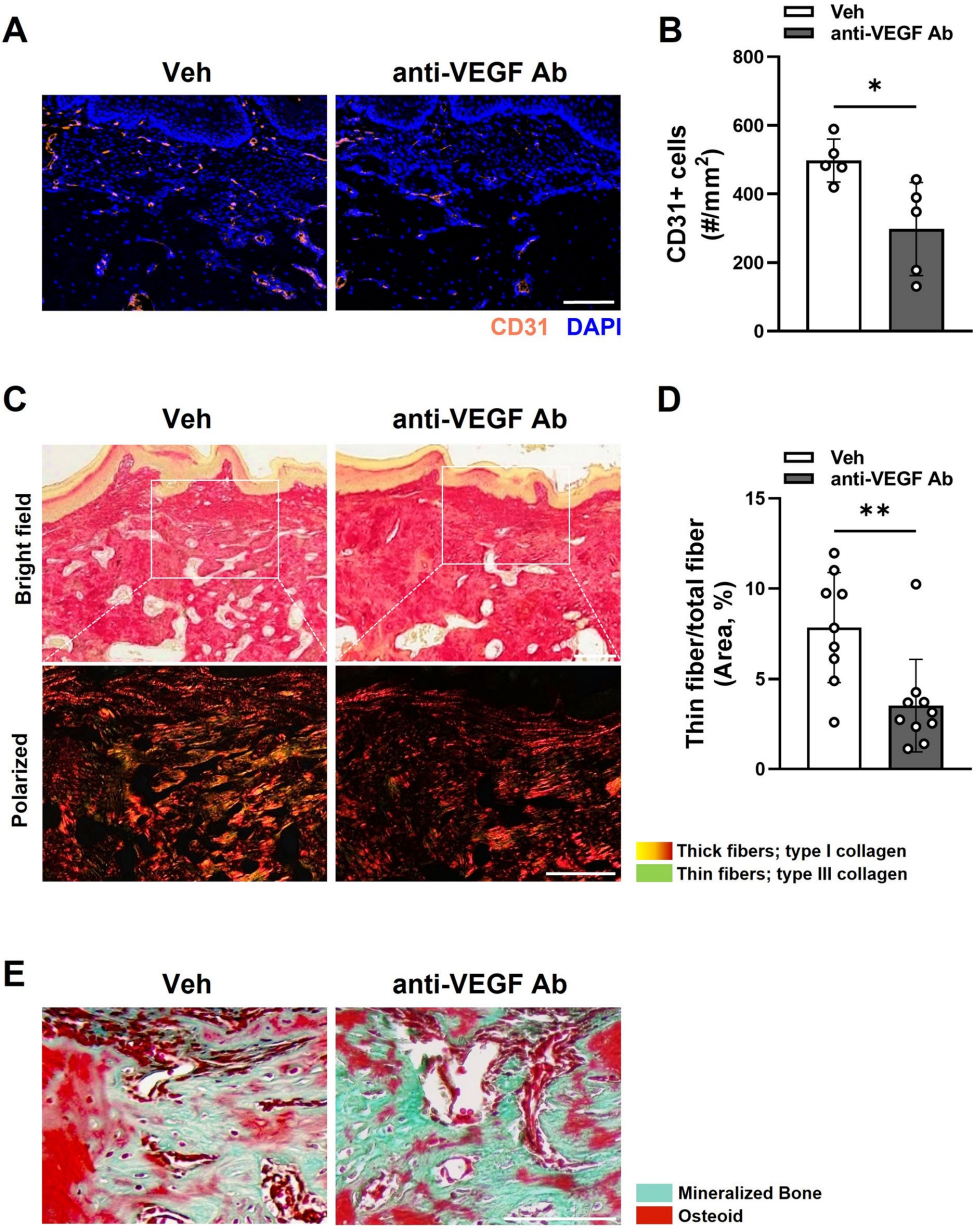
Anti-VEGF Ab causes delayed bone formation by reducing type III collagen expression in the TE mouse model. (**A**) Immunofluorescence staining of CD31 in the tooth extraction sockets. (**B**) Quantification of CD31 + cells. (**C**) Picrosirius Red staining of the tooth extraction sites observed under light or polarized light microscopy. (**D**) Quantification of thin fiber (type III collagen) from the polarized light. (**E**) Masson-Goldner staining from the tooth extraction sites. Scale bar: 100 μm.

To confirm whether anti-VEGF Ab suppressed the vasculature during osteomucosal healing, we performed CD31 immunofluorescent staining and found reduced CD31 expression in the tooth-extraction sockets in the anti-VEGF Ab-treated group (Fig. 2A and B). Collagen formation precedes bone formation during bone healing, and type III collagen is expressed in the early stage of wound healing^19,20^. To examine whether type III collagen has a role in delayed bone healing by anti-VEGF Ab, Picrosirius staining was used to examine type I and type III collagen expression (Fig. 2C). In the tooth extraction sockets, there were diminished type III collagen in anti-VEGF Ab-treated mice (Fig. 2D). Consistent with this finding, the representative images of the Masson-Goldner staining suggest a reduced amount of osteoid in anti-VEGF Ab-treated mice (Fig. 2E), suggesting that suppressed vasculature and reduced type III collagen is associated with delayed bone formation.

### Anti-VEGF Ab does not cause bone necrosis in the TE mouse model

**Fig. 3:**
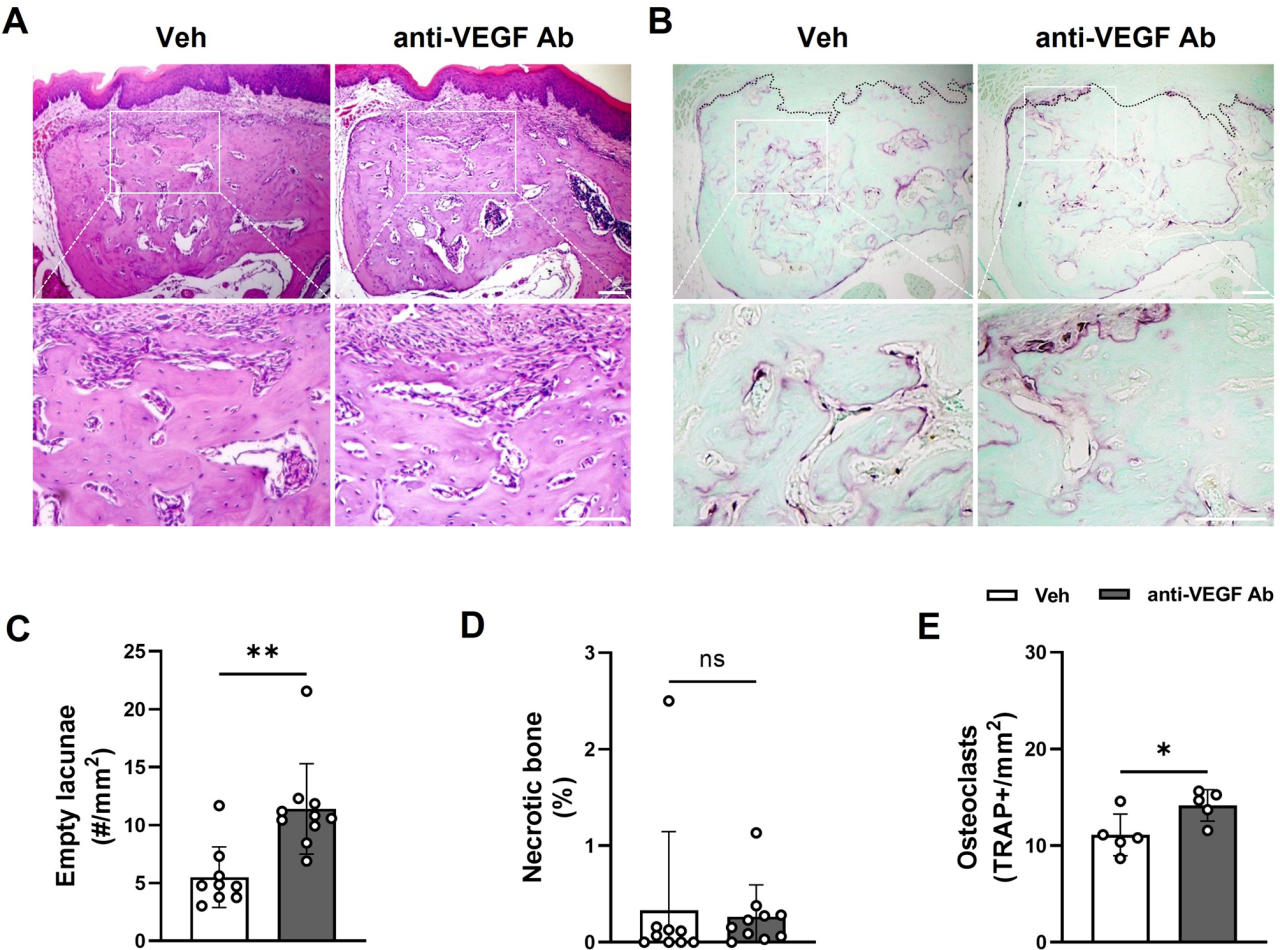
Anti-VEGF Ab does not cause bone necrosis in the TE mouse model. (**A**) H&E staining and (**B**) TRAP staining of the tooth extraction sites in Veh- or anti-VEGF Ab-treated mice. (**C**) Quantification of the empty lacunae and (**D**) necrotic bone area. (**E**) Quantification of TRAP+ osteoclasts. Black dotted line: border between bone and soft tissue. Scale bar: 100 μm.

To determine whether anti-VEGF Ab causes bone necrosis after tooth extraction, H&E-stained sections were evaluated (Fig. 3A). The number of empty lacunae increased in the anti-VEGF Ab-treated mice (Fig. 3C); however, the percentage of the necrotic bone did not show any significant differences (Fig. 3D). On the other hand, an increased number of osteoclast was noted (Fig. 3B and E), suggesting that anti-VEGF Ab did not induce bone necrosis but increased osteoclast formation.

### Anti-VEGF Ab diminished bone formation in the LIP-TE mouse model

**Fig. 4:**
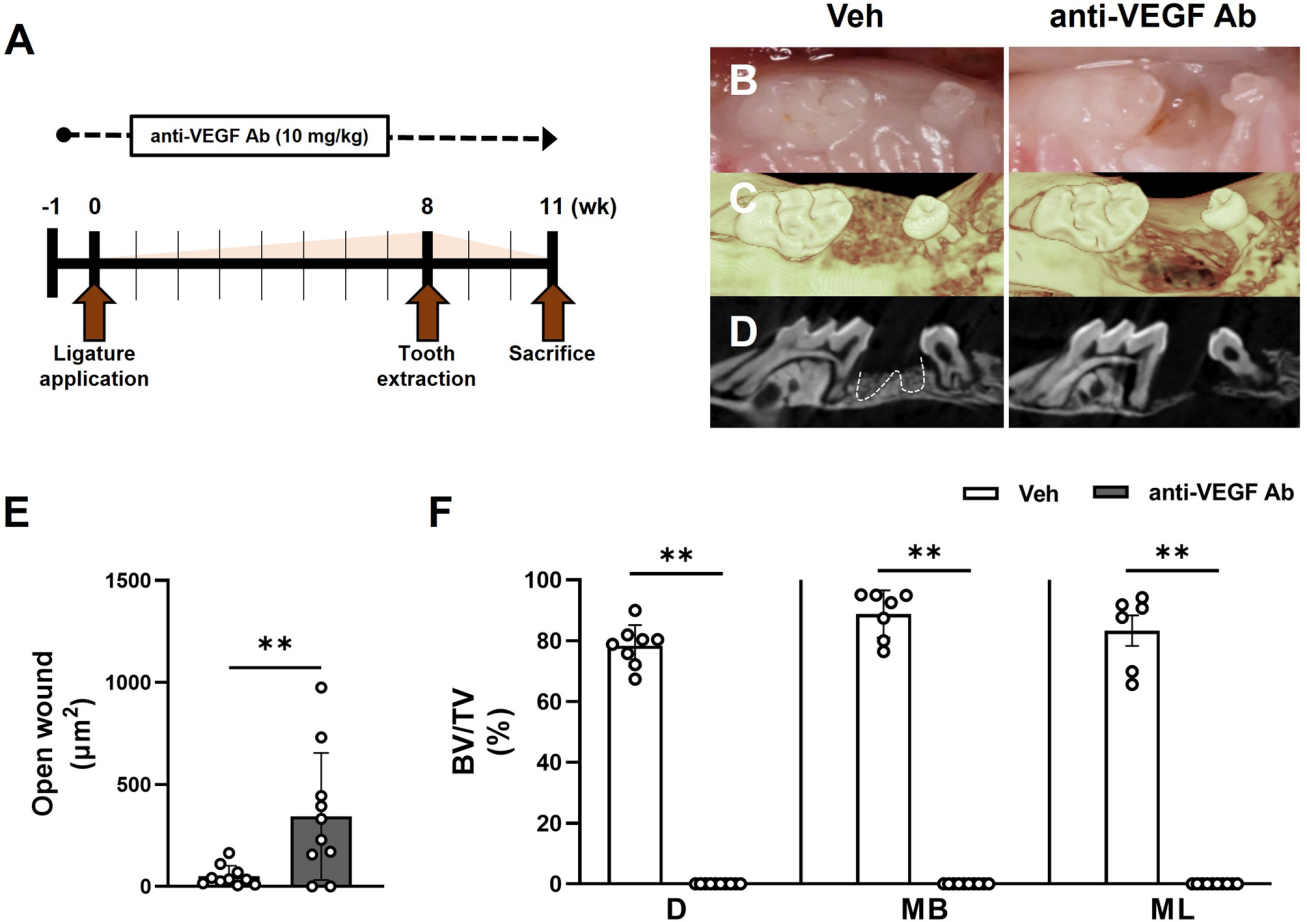
Anti-VEGF Ab diminished bone formation in the LIP-TE mouse model. (**A**) Timeline for LIP-TE model. (**B**) Occlusal views of the clinical photos and (**C**) 3D reconstructed µCT images in Veh- or anti-VEGF Ab-treated mice 3 weeks after extracting both second molars. (**D**) 2D sagittal views of the tooth extraction socket. (**E**) Quantification of the wound closures in the extraction area. (**F**) Quantification of the bone volumes in the distal (D), mesiobuccal (MB), or mesiolingual (ML) root areas following tooth extraction.

A previous study suggested that long-term ligature placement induces chronic inflammation, and tooth extraction after ligature placement exacerbated MRONJ lesions by BPs and Dmab^10^. Using this LIP-TE mouse model, we examined whether anti-VEGF Ab induces osteonecrosis of the jaw (ONJ) lesions. We administered anti-VEGF Ab biweekly to the mice throughout the study period (Fig. 4A). One week after anti-VEGF Ab administration, ligatures were placed around both maxillary second molars. After 8 weeks, the second molars were extracted, and mice were allowed to heal for 3 weeks after which they were euthanized. Unlike the TE mouse model, anti-VEGF Ab-treated mice in the LIP-TE mouse model exhibited a significant increase in the wound defect size (Fig. 4B and E). Also, µCT images (Fig. 4C and D) and BV/TV analyses (Fig. 4F) demonstrated that there was significant inhibition of bone formation in all tooth-extraction sockets in the anti-VEGF Ab-treated mice. These data indicate that anti-VEGF Ab causes delayed soft tissue closure and complete inhibition of bone formation in the presence of chronic inflammation.

### Anti-VEGF Ab caused suppressed vasculature and reduced type III collagen expression in the LIP-TE mouse model

**Fig. 5:**
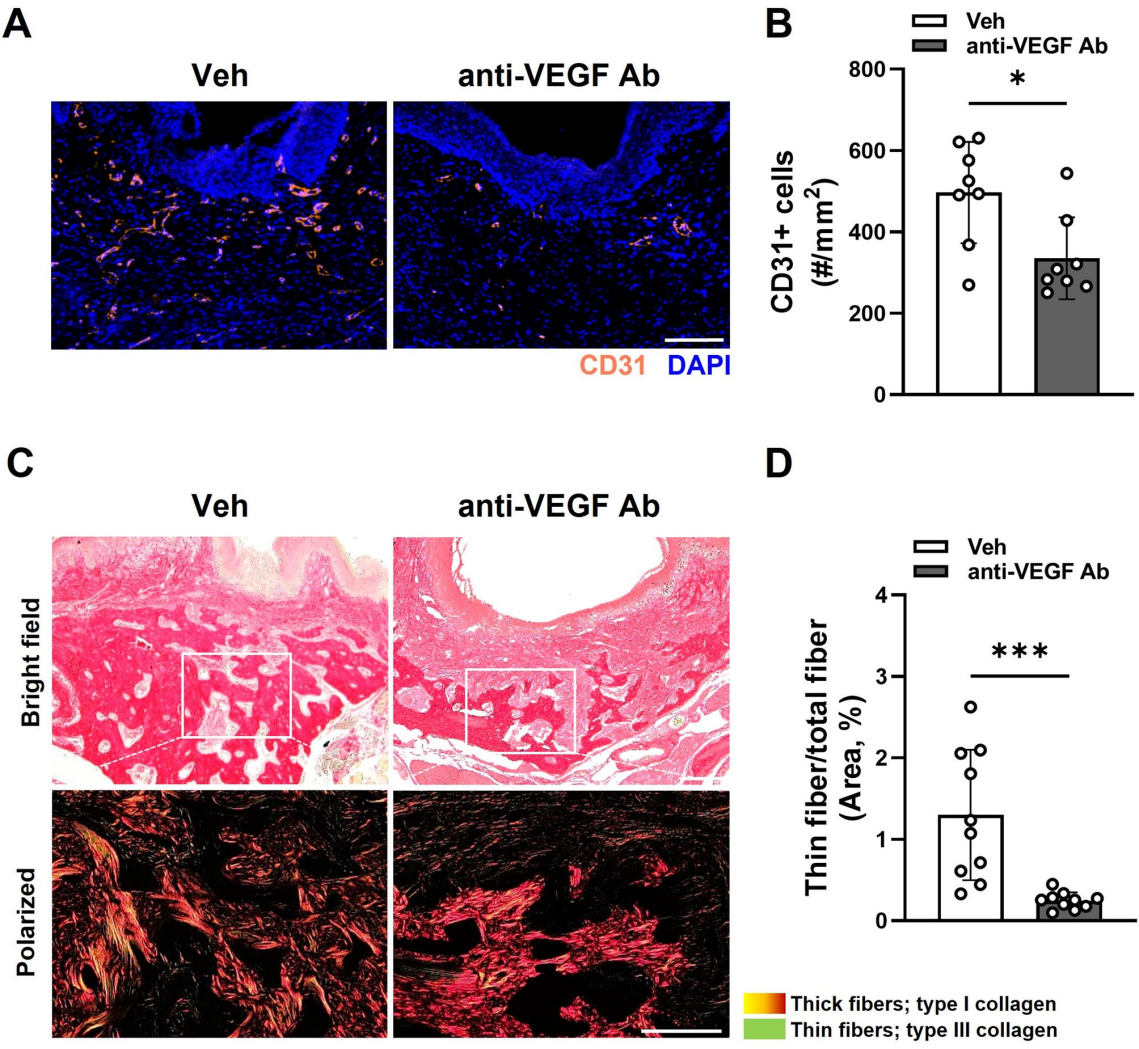
Anti-VEGF Ab causes delayed bone formation by reducing type III collagen expression in the LIP-TE mouse model. (**A**) Immunofluorescence staining of CD31 in the tooth extraction sockets. (**B**) Quantification of CD31 + cells. (**C**) Picrosirius Red staining from the tooth extraction sites observed under the light or polarized light. (**D**) Quantification of thin fiber (type III collagen) from the polarized light. Scale bar: 100 μm.

Similar to the TE mouse model, immunofluorescence staining for the CD31 showed reduced CD31 expression in the tooth-extraction sockets after LIP (Fig. 5A and B). Also, Picrosirius staining demonstrated diminished type III collagen in the anti-VEGF Ab-treated mice (Fig. 5C and D). These data suggest that suppressed vasculature and reduced type III collagen are associated with delayed wound closure and bone formation.

### Anti-VEGF Ab does not cause bone necrosis in the LIP-TE mouse model

**Fig. 6:**
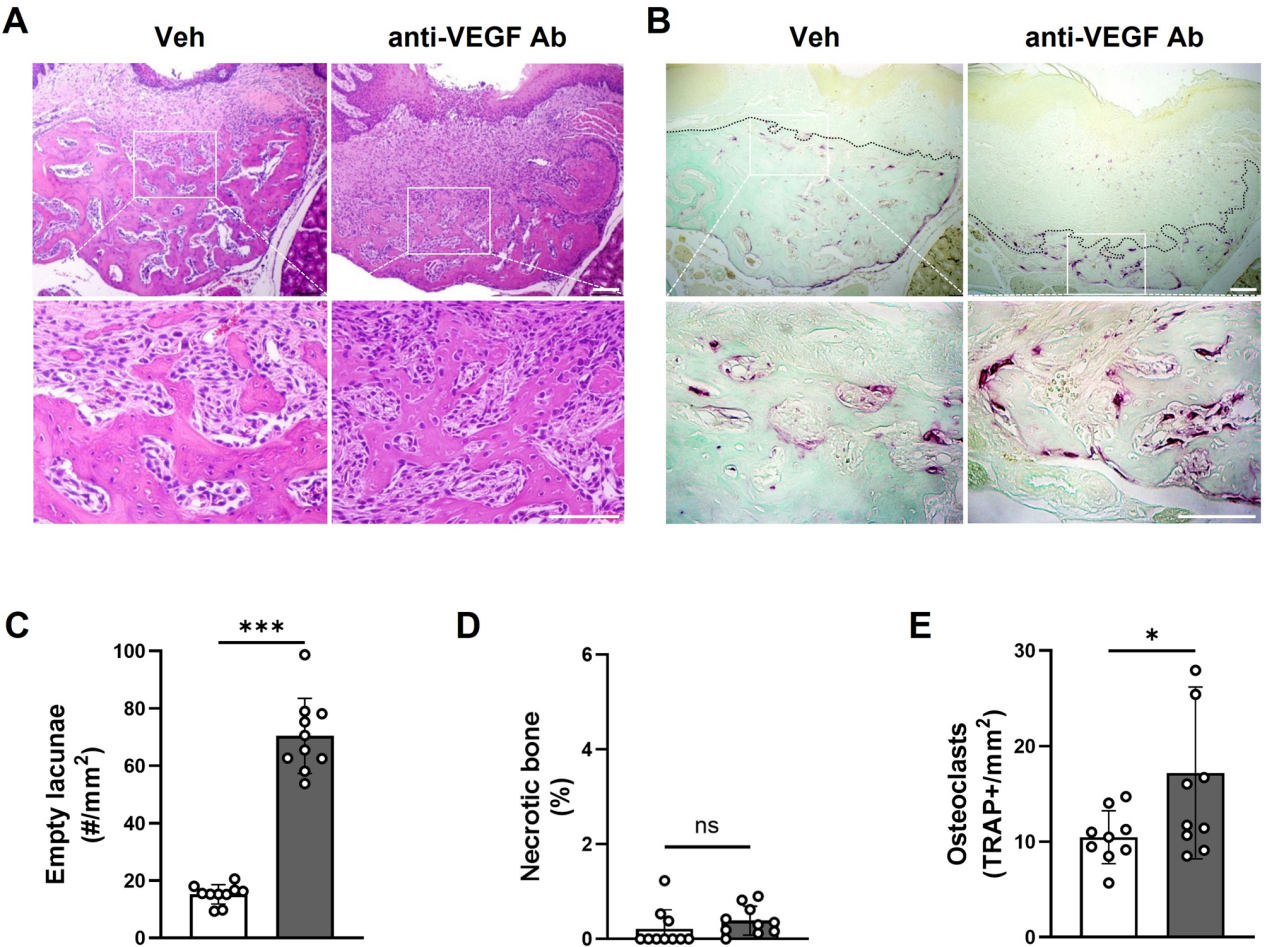
Anti-VEGF Ab does not cause bone necrosis in the LIP-TE mouse model but significantly diminished new bone formation. (**A**) H&E staining and (**B**) TRAP staining of the tooth extraction sites in Veh- or anti-VEGF Ab-treated mice. (**C**) Quantification of the empty lacuna and (**D**) necrotic bone area. (**E**) Quantification of TRAP+ osteoclasts. Black dotted line: border between bone and soft tissue. Scale bar: 100 μm.

To determine whether anti-VEGF Ab causes bone necrosis in the LIP-TE mouse model, H&E-stained sections were evaluated (Fig. 6A). In the anti-VEGF Ab group, the number of empty lacunae increased and distributed throughout the lesions (Fig. 6C); however, there were no clusters of empty lacunae, which are indicative of the necrotic bone (Fig. 6D). Similar to the TE mouse model, the number of osteoclasts increased (Fig. 6B and E). These data suggest that, in the presence of chronic inflammation, anti-VEGF Ab induced severe bone loss and osteoclast formation without causing bone necrosis.

### Delayed osteomucosal healing by anti-VEGF Ab is not associated with T cells in the LIP-TE mouse model

**Fig. 7:**
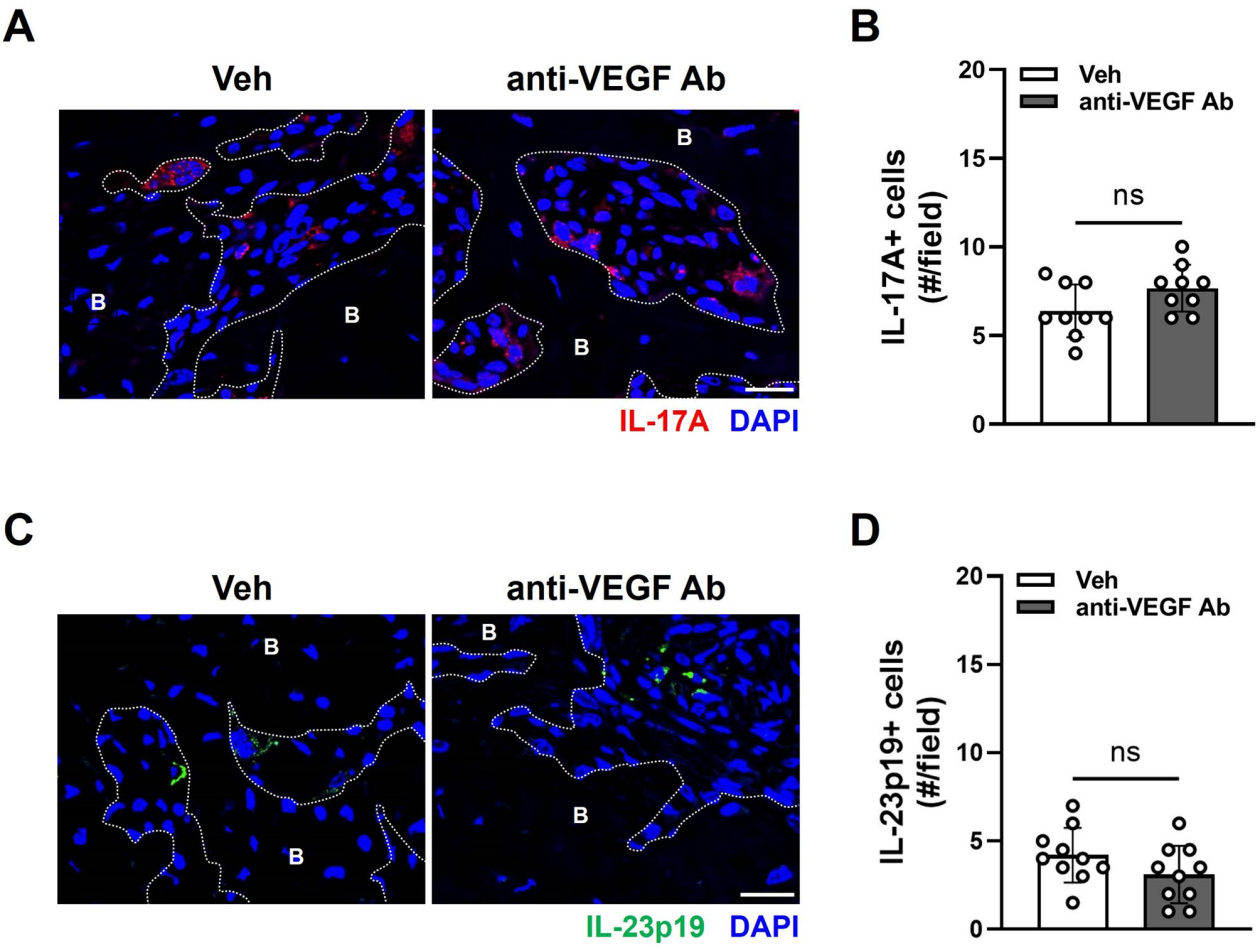
Immunofluorescence staining in the LIP-TE mouse model. (**A**) Immunofluorescent staining of IL-17A in the tooth extraction sockets and (**B**) quantification of IL-17A+ cells. (**C**) Immunofluorescent staining of IL-23p19 in the tooth extraction sockets and (**D**) quantification of IL-23p19+ cells. B: bone area. Scale bar: 20 μm.

Previously, IL-17, a pro-inflammatory cytokine produced by Th17 cells, was found to be elevated in patients with osteonecrosis of the femoral head and in mice with MRONJ by BPs^21,22^. IL-23 is known to be critical for generation of Th17 cells^23^. To determine whether the delay in wound healing by anti-VEGF Ab is associated with Th17 cells, immunofluorescence staining was performed for IL-17 and IL-23 (Fig. 7). Unlike MRONJ lesions by BPs, there were no changes in the numbers of IL-17-producing cells (Fig. 7A and B) and IL-23-producing cells (Fig. 7C and D) between Veh- and anti-VEGF Ab-treated mice. These data indicate that Th17 cells are not associated with anti-VEGF Ab-mediated delayed osteomucosal healing.

## Discussion

Bmab (anti-VEGF Ab) is widely used to reduce cancer burdens^15^, and increasing lines of evidence suggest that it delays wound healing in humans^24^. Indeed, Bmab has been shown to be associated with MRONJ development^11–14^, but its precise role in osteomucosal healing in the oral cavity in the context of MRONJ is unclear. In this study, we used both TE and TE-LIP mouse models to evaluate the effect of anti-VEGF neutralizing antibody on osteomucosal healing in mice following tooth extraction, with or without chronic inflammatory conditions. Our study demonstrated that anti-VEGF Ab suppressed both soft and hard tissue healing by inhibiting angiogenesis and collagen formation. More importantly, anti-VEGF Ab alone did not develop bone necrosis, suggesting that anti-VEGF Ab is not directly associated with MRONJ.

In the TE mouse model, anti-VEGF Ab significantly reduced new bone formation and type III collagen formation in the extracted socket. However, it alone did not cause any soft tissue defects after tooth extraction. Similarly, Akita et al. reported that anti-VEGFA mAb therapy delayed osseous but not soft tissue healing following tooth extraction^11^. It may be attributed to short-term administration of anti-VEGF Ab without any chronic inflammatory conditions.

On the other hand, when chronic pre-existing inflammatory condition was introduced using LIP, anti-VEGF Ab severely dampened the osteomucosal healing process including defects in soft tissue healing, suppressed bone formation, increased bone resorption, and decreased type III collagen production. Messer et al. previously showed that anti-VEGF mAb caused a destructive advanced form of periodontitis without bone necrosis in rice rats^18^. Because rice rats spontaneously develop localized periodontitis, chronic inflammatory conditions such as periodontitis may play a critical role in exacerbating delayed healing by anti-VEGF Ab.

Although anti-VEGF Ab suppressed angiogenesis (Figs. 2A and B and 5A and B), it also inhibited collagen synthesis (Figs. 2C and D and 5C and D). VEGF is known to induce differentiation of endothelial cells, but it also induces collagen production by fibroblasts^25,26^. In osteomucosal healing, proper formation of provisional collagen matrixes is the key to success in complete healing of both soft and hard tissues^2^. In soft tissue healing, Type III collagen is expressed in the early stage of wound healing^27^, and the epithelial cells migrate onto the provisional collagen matrixes to close the wound. For the hard tissue healing, deposition of collagens is an indispensable step in the early stages of woven bone formation because mineralization occurs by nucleation process^28^. Therefore, anti-VEGF Ab may have a dual role in delaying wound healing by inhibiting both blood vessel and connective tissue formation.

Monocyte/macrophage lineage cells, including osteoclasts, express VEGF receptors^29^, and VEGF has been shown to promote the survival and differentiation of osteoclasts^30,31^. But anti-VEGF Ab did not inhibit osteoclast differentiation in this study. In fact, prolonged treatment of anti-VEGF Ab in the presence of LIP increased the numbers of osteoclasts, and these osteoclasts seem to be functional as demonstrated by increased bone resorption. This is in sharp contrast to the MRONJ lesions by bisphosphonates or anti-RANKL Ab in which osteoclasts are either dysfunctional or absent, respectively, leading to non-resorbing bone even in the presence of severe inflammatory conditions^2^. This notion further validates clinical observations that bone necrosis is uniquely associated with osteoclast-targeting medications such as BPs and Dmab.

It is worth noting that, while the numbers of empty lacunae were increased by anti-VEGF Ab in both models, there were no changes in the bone necrosis (Figs. 3 and 6). By definition, bone necrosis is histologically determined by the clusters of ≥ 5 contiguous empty lacunae^3,32,33^. It is then conceivable that inhibition of blood vessel formation by anti-VEGF Ab caused no blood supply to the osteocytes, continuously leading to an increased cell death and empty lacunae. On the other hand, anti-VEGF Ab did not interfere with osteoclast differentiation and functions; instead, the number of osteoclasts were increased by anti-VEGF Ab. As such, bone with empty lacunae was effectively removed by osteoclasts, causing no changes in the bone necrosis. Because osteoclasts were not affected by anti-VEGF Ab and no bone necrosis was observed, these data indicate that anti-VEGF Ab alone does not induce MRONJ.

Immunofluorescence staining against IL-17 and IL-23 showed that anti-VEGF Ab has no effect on the expression of IL-17 and IL-23 around the tooth-extraction sockets despite delayed osteomucosal wound healing (Fig. 7). Th17 cells have been shown to be increased in MRONJ lesions^10^. The fact that IL-17 and IL-23 did not change in anti-VEGF Ab-treated mice implies that Th17 cells are not associated with the delayed healing process by anti-VEGF Ab.

ORN has similar clinical presentations to MRONJ such as exposed necrotic bone and impaired wound healing but occurs due to radiation therapy^34^. The lack of blood supply to bone cells such as osteocytes is one of the main causes of bone necrosis by ORN^35^. For this reason, it has been speculated that anti-VEGF Ab can also cause bone necrosis. However, it is noteworthy to distinguish the pathophysiology of MRONJ from that of ORN. Radiation destroys local existing structures in their radiation path by generating free radicals that damage the cells within, leading to permanent tissue alterations such as muscle atrophy, fibrosis, and blood vessel degeneration^36^.

Anti-VEGF Ab, on the other hand, primarily affects the newly forming blood vessels and not the already-formed existing blood vessels because it targets VEGF^17^. This notion has several important implications. First, blood supply to the osteocytes already embedded in the existing bone will not be affected by anti-VEGF Ab. As such, no bone necrosis is observed. Second, only the wounded site is affected by anti-VEGF Ab because it blocks new blood vessel formation in the wounded site, leading to delayed healing. Lastly, unlike radiation, anti-VEGF Ab will not permanently alter intrinsic characteristics of cells, suggesting that damages by anti-VEGF Ab are reversible. For these reasons, different clinical presentations by anti-VEGF Ab are anticipated from that of radiation therapy.

## Conclusion

This study demonstrates that anti-VEGF Ab does not induce bone necrosis but delays osteomucosal wound healing by suppressing neovascularization and collagen formation in the tooth extracted sites. Because many clinical reports have shown that anti-VEGF Ab is associated with MRONJ lesions with additional medications, future studies should investigate possible additive effects with other medications in exacerbating MRONJ lesions.

## Materials and methods

### Animals

Forty 8-week-old female C57BL/6 mice were purchased from the Jackson Laboratory (Bar Harbor, ME, USA) and adapted for one week at animal facilities in the Division of Laboratory Animal Medicine of University of California Los Angeles. The experimental protocol of this study was approved by the Chancellor’s Animal Research Committee (ARC-2022-012), and this study strictly followed recommendations listed on the Guide for the Care and Use of Laboratory Animals of the National Institutes of Health. All animal methods were performed in accordance with the relevant guidelines and regulations. This study is reported in accordance with the ARRIVE guidelines.

### Study model

During the study period, mice were administrated intraperitoneally twice per week with either an anti-VEGF Ab (10 mg/kg; murine anti-VEGF monoclonal antibody, B20-4.1.1, Genentech, South San Francisco, CA) (anti-VEGF Ab-treated group) or sterile saline (Veh-treated group). For the TE mouse model, both maxillary first molars were atraumatically extracted after one week of initial administration (*n* = 10 per group). For LIP-TE mouse model, a 5-0 silk ligature was placed around both maxillary second molars to induce periodontitis after one week of initial administration (*n* = 10/group). Eight weeks after the ligature induction of periodontitis, the ligated molars were atraumatically extracted. The ligature placement and extraction were performed under general anesthesia using a ketamine (100 mg/kg) and xylazine (5 mg/kg) cocktail. For both mouse models, the mice were euthanized after 3 weeks of tooth extraction using CO_2_ gas and subjected to occlusal photography to evaluate wound closure. The maxillae were then fixed overnight in 4% paraformaldehyde (PFA) solution at 4 °C, subsequently washed in PBS, and stored in 70% ethanol for micro-computed tomography (µCT) scanning.

### µCT scan and volumetric analysis

To volumetrically examine bone reconstruction within the extraction sockets, the maxillae were scanned using a µCT scanner (SkyScan 1275; Bruker, Kontich, Belgium). The scanning parameters were set to a voxel size of 10 µm^3^, using a 1.0 mm aluminum filter at 60 kVp and 166 µA. The acquired images were reconstructed into 3D datasets using NRecon (Bruker). Bone volume fraction (BV/TV, %) was measured for each root using CTAn software (Bruker): in the TE model (first molar), the distolingual, distobuccal, and mesial roots; and in the LIP-TE model (second molar), the distal, mesiobuccal, and mesiolingual roots. The region of interest (ROI) was defined from the cementoenamel junction (CEJ) of the adjacent tooth to a point 150 μm above the maxillary sinus. For ROI selection, a global thresholding approach was applied (gray-scale threshold range: 100–225), with unmineralized osteoid excluded from the calculation.

### Histological and histomorphometric analysis

EDTA-decalcified tissues were sectioned in the coronal plane with a thickness of 5 μm using a rotary microtome (Microm HM355S; Thermo Fisher Scientific, Walldorf, Germany). For each histochemical staining, four tissue sections spaced 50 μm apart were selected per sample. Images of the stained sections were obtained with an optical microscope (Olympus CKX41; Olympus, PA, USA) equipped with a digital camera (Olympus DP-72; Olympus) using 10× and 20× objectives. All the histomorphometry was performed by a blinded and calibrated investigator by using the Image J software (ImageJ 1.53t; National Institutes of Health, Bethesda, MD, USA).

### Quantification of empty lacunae and necrotic bone

H&E staining was performed to visualize lacunae and osteocytes. Empty lacunae were histologically identified by the absence of an osteocyte nucleus, and the number of empty lacunae was divided by the total bone area. For quantifying necrotic bone, regions with empty lacunae clusters (five or more empty lacunae)^3,32,33^ were measured and divided by the total bone area.

### Quantification of osteoclasts

To identify tartrate-resistant acid phosphatase (TRAP) in osteoclasts, TRAP staining was performed according to the manufacturer’s protocol (387 A; Sigma-Aldrich, St. Louis, MO, USA). Briefly, deparaffinized slides were rehydrated through graded ethanol to distilled water. The slides were placed in pre-warmed TRAP staining solution and incubated at 37 ℃ for 30 min in the dark and were subsequently counterstained with 0.02% Fast green for 5 min. TRAP-positive multinucleated cells (> 5 nuclei) were counted as osteoclasts.

### Picrosirius-polarization method for birefringent fibers

To analyze collagen fibers within the extracted alveolar socket, sections were stained using Picrosirius Red Stain Kit (ab150681; Abcam, Cambridge, UK). The birefringence of fibers was visualized under bright-field and polarized-light microscopy. Picrosirius-polarization method was used for the assessment of birefringent fibers, categorized as red or yellow-stained type I collagen (thick fibers) and green‐stained type III collagen (thin fibers)^20^. The region of interest (ROI) was set as 400 µm^2^ from the top-middle of the new bone within the socket, and the color threshold of each collagen type was selected by Lab color space.

### Masson-Goldner staining

To visualize the connective tissue and determine old mineralized and newly generated osteoid tissues, the tissue sections of the TE model mice were stained with Masson-Goldner staining using the supplier’s protocol (1.00485.001; Sigma-Aldrich).

### Immunofluorescence staining

Tissue sections were dehydrated through a graded ethanol series and subsequent antigen unmasking was carried out in citric acid-based antigen unmasking solution (Vector Laboratories, Burlingame, CA, USA) at 98 °C for 20 min. The sections were blocked using 2% bovine serum albumin (BSA) and animal-free blocker (Vector Laboratories) in a 1:1 ratio for 1 h and incubated overnight with primary antibodies at 4 °C. The following primary antibodies were used: CD31 (ab222783; Abcam), IL-17A (ab79056; Abcam), IL-23p19 (AF1619; Abcam). Afterward, secondary antibodies were applied for 1 h and the slides were mounted with a DAPI-containing mounting medium (Vectashield Vibrance; Vector Laboratories). Fluorescence imaging was conducted using a confocal microscope (LSM700; Carl Zeiss Microscopy, Inc., Dublin, CA, USA).

### Statistical analysis

To determine the difference between groups, the student t-test was performed using statistical program (Prizm 9; GraphPad, San Diego, CA, USA). A *p*-value < 0.05 indicated a significant difference between groups.

## Data Availability

The datasets generated during and/or analyzed during the current study are available from the corresponding author on reasonable request.
